# Supplementary material for: High sporulation and overexpression of virulence factors in biofilms and reduced susceptibility to vancomycin and linezolid in recurrent Clostridium [Clostridioides] difficile infection isolates
Source: PLoS One. 2019 Jul 31;14(7):e0220671. doi: 10.1371/journal.pone.0220671 (PMC6668830; doi:10.1371/journal.pone.0220671)
Supplement: Attachment — Submitted filename: Response to Reviewers.doc [file pone.0220671.s001.doc]

Yung-Fu Chang

Academic Editor

*PLOS One*

July 3, 2019

Dear Dr. Chang,

Please find the corrections and responses to the comments kindly given to the manuscript entitled “High sporulation and overexpression of virulence factors in biofilms and reduced susceptibility to vancomycin and linezolid in recurrent *Clostridium* [*Clostridioides*] *difficile* infection isolates” that we submitted to the *PLOS One.*

Also, all the authors of this study declare that all relevant data underlying our study's findings can be found within the manuscript.

We thank the editor and reviewers for their comments and hope that the revisions will help clarify the manuscript. We believe that the manuscript has been strengthened as a result of this process.

Thank you for considering this work.

Elvira Garza-González

Avenida Madero s/n Colonia Mitras Centro

Monterrey, Nuevo Leon, 64460, Mexico

Phone: +52 (81) 83 29 41 66

Fax +52 (81) 83 29 41 66.

E-mail: [elvira_garza_gzz@yahoo.com](mailto:elvira_garza_gzz@yahoo.com)

Reviewer #1:

**1) line 300: minimum inhibitory concentration of the biofilm was determined in C diff bioflm model or mocrobiota-Cdiff biofim model?**

**Response:** The minimum inhibitory concentrations were determined from *C. difficile* biofilm without microbiota. The point was clarified in the new manuscript in line 301.

**2) It would be more informative to check transcription of toxins, germination of spores, and adhesion and biofilm formation capability.**

**Response:** Regarding the adhesion and biofilm formation, we calculated the adherence capability as adherence index for each isolate producing biofilm, and there was no difference between R-CDI and NR-CDI. This information is included in the manuscript.

We added transcription of toxins as perspectives of the study (line 336). However, we expect a low impact because toxin transcription has shown to be ribotype-dependent and our population was mostly infected with 027 ribotype in either R-CDI or NR-CDI isolates.

We also added germination of spores as perspective (line 330) because our data support the role of sporulation pathway genes *(sigH*, *spo0A*), in the development of recurrent CDI.
